# Effect of Alumina on Crystallization Behavior of Calcium Ferrite in Fe_2_O_3_-CaO-SiO_2_-Al_2_O_3_ System

**DOI:** 10.3390/ma15155257

**Published:** 2022-07-29

**Authors:** Rui-Feng Xin, Yu Du, Xing-Min Guo

**Affiliations:** State Key Laboratory of Advanced Metallurgy and School of Metallurgical and Ecological Engineering, University of Science and Technology Beijing, Beijing 100083, China; xinrf@hotmail.com

**Keywords:** sinter, binding phase, calcium ferrite, crystallization, alumina

## Abstract

Al_2_O_3_ is a gangue component in iron ores, significantly influencing the formation and crystallization of calcium ferrite in the sintering process. But the mechanism of the Al_2_O_3_ effect on the crystallization of calcium ferrite is rarely reported. In this work, a crystallization device was designed to investigate the crystallization behavior of calcium ferrite in Fe_2_O_3_-CaO-SiO_2_-Al_2_O_3_ melt under non-isothermal conditions. XRD, SEM-EDS, and optical microscopy were used to identify the crystalline phase and the microstructure of samples. The result shows that the crystal morphology of SFCA changed in the order of strip, column, and needle as the Al_2_O_3_ content increased. The crystallization sequence of samples containing Al_2_O_3_ was observed as Ca_4_Fe_14_O_25_ (C_4_F_14_) → Fe_2_O_3_ → Ca_3.18_Fe_15.48_Al_1.34_O_36_ (SFCA-I) → CaFe_2_O_4_ (CF) → Ca_5_Si_2_(Fe, Al)_18_O_36_ (SFCA) → γ-Ca_2_SiO_4_ (C_2_S). The generation pathway of SFCA-I was found to be C_4_F_14_ + Si^4+^ + Al^3+^ → SFCA-I. Increasing the cooling rate can promote the formation of C_4_F_14_, SFCA-I, Fe_2_O_3_ and the amorphous phase. However, it prevented the crystallization of CF and SFCA while inhibiting the transformation of β-C_2_S to γ-C_2_S. When the Al_2_O_3_ content reached or exceeded 2.5 mass pct, the viscosity of Fe_2_O_3_-CaO-SiO_2_-Al_2_O_3_ melt increased sharply, resulting in the decrease in the crystal size of calcium ferrite.

## 1. Introduction

High-basicity sinter is mainly utilized as a critical iron-containing material for blast furnace ironmaking, where calcium ferrite is the predominant binding phase [1,2]. The mineral composition and microstructure of the binding phase has an important influence on the quality of the sinter [3,4,5]. Most of the binding phase is mainly complex calcium ferrite.

Recently, with the increasing consumption of high-alumina iron ores, the investigations focused on the role of Al_2_O_3_ in the formation and crystallization of the binding phase have increased substantially [6,7,8]. Researchers [9,10,11] have found that adding a moderate quantity of Al_2_O_3_ can promote the formation of complex calcium ferrite.

In sinter, some studies [12,13,14,15] revealed two primary crystal forms of complex calcium ferrite as SFCA (Ca_5_Si_2_(Fe, Al)_18_O_36_) and SFCA-I (Ca_3.18_Fe_15.48_Al_1.34_O_36_). Compared to SFCA (column and lath), needle-shaped SFCA-I is more favorable for releasing internal stress to improve the strength of the sinter [16]. Furthermore, the microstructure and morphology of complex calcium ferrite also have an important influence on the strength of sinter [17]. Webster et al. [18] investigated the effect of Al_2_O_3_ on the formation process and thermodynamic stability of complex calcium ferrite. Liles et al. [19] investigated SFCA using the structural refinement approach, finding that Fe^3+^, Si^4+^, and Al^3+^ tended to occupy the tetrahedral positions of SFCA, while Fe^3+^, Ca^2+^ in the octahedral locations. The ion replacement is 2(Fe^3+^, Al^3+^) = Ca^2+^ + Si^4+^ on electric neutrality. In addition, lowering the temperature is aided in replacing Al^3+^ ↔ Fe^3+^.

Ding et al. [20] studied the crystallization kinetics of the CaO-Fe_2_O_3_ binary system by the DSC method using Avrami and Mo models. In addition, the crystalline surface activation energies of Ca_2_Fe_2_O_5_ (C_2_F) and CaFe_2_O_4_ (CF) were calculated using the Kissinger model. It was found that the crystallization rate of C_2_F is faster than that of CF, while when the cooling rate increases, the crystallization of CF is accelerated, where inversely the crystallization of C_2_F is inhibited. Yang et al. [21] investigated the role of Al_2_O_3_ in the crystallization behavior of the Fe_2_O_3_-CaO-Al_2_O_3_ melt during the cooling process. It was found that adding a tiny quantity of Al_2_O_3_ can improve the preferred orientation of solid solution in CaFe_2_O_4_, at the same time significantly affecting the fracture toughness of the sample. Park et al. [22] revealed the influence of Al_2_O_3_ on the reduction performance of the Fe_2_O_3_-CaO-Al_2_O_3_-SiO_2_ pseudo-quaternary system through in situ observation by high temperature confocal laser scanning micro-scope, finding that the increase of Al_2_O_3_ led to the crystallization of Fe_2_O_3_. With the increase of cooling rate, the crystallization of SFCA was promoted, and the reducibility was improved.

In this study, the crystallization mechanism of Al_2_O_3_ on the Fe_2_O_3_-CaO-SiO_2_-Al_2_O_3_ system was examined in the non-equilibrium state. A crystallization device was introduced where the cooling rate can be precisely measured, and this approach may assure that the crystallization morphology and composition of the sample are more realistic. The finding of this study is benefit to understand the phase transition of the Fe_2_O_3_-CaO-SiO_2_-Al_2_O_3_ system during the crystallization process. It lays a foundation for promoting the crystallization of SFCA to improve the quality of sinter.

## 2. Experimental Procedures

### 2.1. Preparation of Samples

Analytical grade reagents of Fe_2_O_3_ (≥99.9 pct, Sinopharm Chemical Reagent Co., Ltd., Shanghai, China), CaCO_3_ (≥99.5 pct, Sinopharm Chemical Reagent Co., Ltd., Shanghai, China), SiO_2_ (≥99.9 pct, Sinopharm Chemical Reagent Co., Ltd., Shanghai, China), and Al_2_O_3_ (≥99.9 pct, Sinopharm Chemical Reagent Co., Ltd., Shanghai, China) were used to prepare the samples. The chemical composition of the initial samples is shown in Table 1. A study revealed that [23], when the molar ratio of Fe_2_O_3_ to CaO in the binding phase is in the range of 1.25 to 1.59 in the actual sintering process, more liquid phase and the acicular-shape, column-shaped or columnar calcium ferrite would be generated. Therefore, the Fe_2_O_3_ to CaO molar ratio in this study was set at 1.3. In addition, 4.0 mass pct SiO_2_ was chosen as the gangue in the iron ores, with Al_2_O_3_ content range from 0 to 3.0 mass pct. To ensure that the entire crystallization process was carried out in the pure liquid phase, the liquidus temperature (LT) of each sample was calculated using FactSage 8.2 software [24].

### 2.2. Sinter Process

At room temperature, Fe_2_O_3_, CaO, SiO_2_, and Al_2_O_3_ were mixed evenly, as stated in Table 1. For improving precision, CaCO_3_ was used to replace CaO with an equal-molar quantity for precise weighing. 20.0 g of sample and an appropriate amount of anhydrous ethanol (≥99.7 pct, Sinopharm Chemical Reagent Co., Ltd.) were mixed evenly, then roasted at 200 °C for 3 h in a drying oven under an air atmosphere. The sample was compressed into a cylindrical shape (Ø 20 × 20 mm) and sintered in a platinum crucible. From the previous research [25,26,27] it was found that if the sample was held above the TL for 2 h, a molten equilibrium liquid phase would be formed.

**Figure 1:**
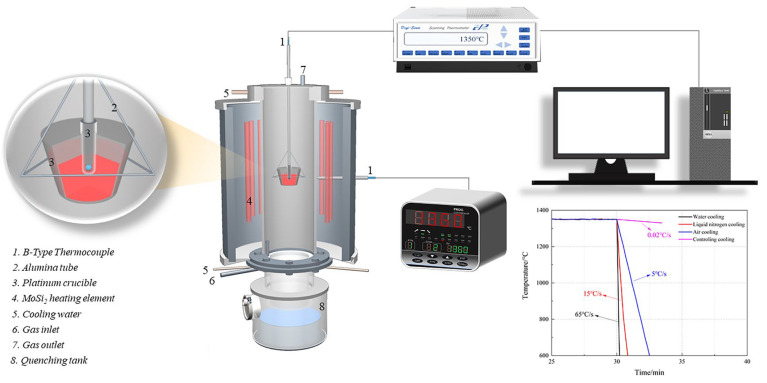
Schematic diagram of cooling crystallization device.

In this experiment, in order to obtain a complete equilibrium liquid phase, the sample was heated to 1350 °C at a heating rate of 5 °C/min and held for 4 h in air atmosphere. Subsequently, the samples were treated under the condition of various cooling rates (0.02 °C/s, 5 °C/s, 15 °C/s, and 65 °C/s.) [28] as presented in Figure 1.

For obtaining the order of different crystallization phases, once the samples were cooled to the target temperature at a cooling rate of 0.02 °C/s, water cooling was conducted to obtain an instantaneous mineral composition at the corresponding temperature.

### 2.3. Phase Determination

A part in each sample was ground to a particle size of less than 50 μm passing through the sieve completely for XRD determination. The mineral phase of the crystalline powder samples was identified using a Rigaku SmartLab X-ray diffractometer (Rigaku Corporation, Tokyo, Japan). Cu Kα was used as the radiation source (40 kV, 150 mA) with a graphite curved monochromator in the diffracted beam path. The wavelength is 0.15406 nm, with a scanning speed of 10°/min, a scanning step length of 0.02°, and a scanning range (2θ) from 10° to 100°. XRD data were matched using Crystallographica Search-Match software (CSM3.0, Oxford Cryosystems Ltd., UK, Oxford).

The other part of the samples was embedded into the ethylenediamine-doping epoxy resin and polished for the microstructure observation. The mineral morphology and structure were observed by optical microscope (Optical Instrument Fifth Factory Co., Ltd., Shanghai, China) and scanning electron microscope (Zeiss GeminiSEM500, Berlin, Germany). The device is equipped with EDS (Ultim Max 170, Berlin, Germany) to detect elemental composition.

## 3. Results and Discussion

### 3.1. Effect of Al_2_O_3_

**Figure 2:**
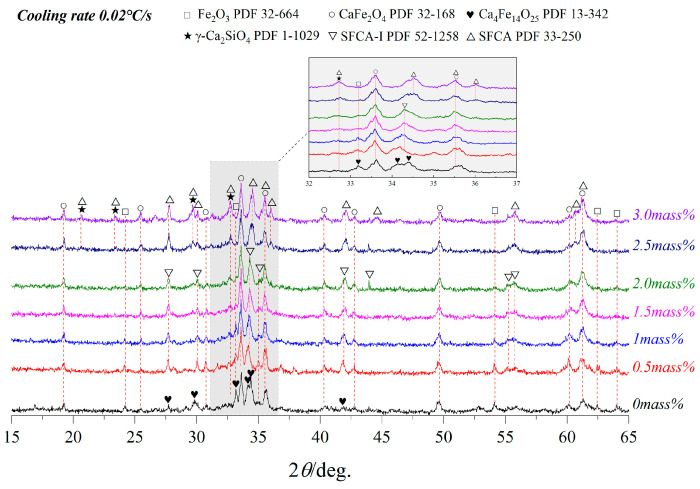
XRD patterns of samples cooled at 0.02 °C/s.

**Figure 3:**
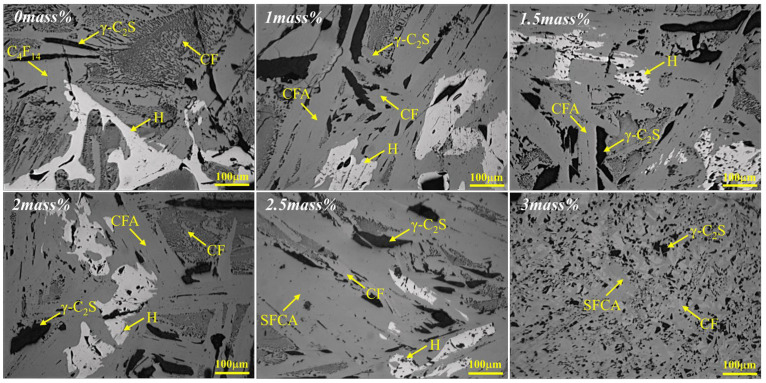
Optical photos of the samples cooled at 0.02 °C/s (CF: CaFe_2_O_4_; C_4_F_14_: Ca_4_Fe_14_O_25_; SFCA-I (CFA): Ca_3.18_Fe_15.48_Al_1.34_O_36_; SFCA: Ca_5_Si_2_(Fe, Al)_18_O_36_; γ-C_2_S: γ-Ca_2_SiO_4_; H: Fe_2_O_3_).

**Figure 4:**
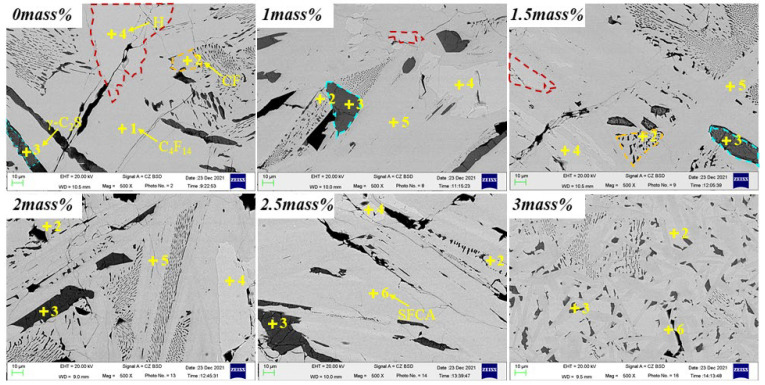
SEM photos of the samples cooled at 0.02 °C/s.

The composition change of the sample in equilibrium cooling process was obtained by heating to 1350 °C for 4 h and then cooling to room temperature at a rate of 0.02 °C/s. Figure 2, Figure 3 and Figure 4 and Table 2 depicts the XRD patterns, optical micrograph, SEM, and EDS results of the crystalline samples containing varying amounts of Al_2_O_3_, respectively.

It is indicated that Ca_4_Fe_14_O_25_(C_4_F_14_), CF, γ-Ca_2_SiO_4_(γ-C_2_S), SFCA-I, SFCA and Fe_2_O_3_ were crystallized. The increase of Al_2_O_3_ content led to the gradual decreases of C_4_F_14_, Fe_2_O_3_, and γ-C_2_S. SFCA-I first increased and then decreased. CF and SFCA increased gradually. The detailed result is as follows:

(1) When Al_2_O_3_ was not added, Ca^2+^ reacted with Fe^3+^ and O^2−^ to form C_4_F_14_ and CF, while Ca^2+^ reacted with Si^4+^ and O^2−^ to form γ-C_2_S;
(2) When Al_2_O_3_ reached 0.5 mass pct, C_4_F_14_ disappeared, and CF had gradually increased, indicating that the preferentially crystallized C_4_F_14_ reacted with Al^3+^ and Si^4+^ to form SFCA-I;
(3) When Al_2_O_3_ reached 2.0 mass pct, CF and Fe_2_O_3_ had gradually decreased, γ-C_2_S had not changed significantly, and SFCA-I increased gradually. It shows that CF also participated in the generation of SFCA-I.
(4) When Al_2_O_3_ reached 2.5 mass pct, the iron-rich SFCA-I was transformed into SFCA (high Si, high Al). Simultaneously, it promoted the precipitation of Fe_2_O_3_. Fe_2_O_3_ and CF increased, and Si^4+^ was mainly involved in generating SFCA, resulting in the decrease of γ-C_2_S.
(5) When Al_2_O_3_ reached 3.0 mass pct, CF and SFCA continued to increase, while γ-C_2_S decreased and Fe_2_O_3_ disappeared.

### 3.2. The Sequence of Crystallization Phase

**Figure 5:**
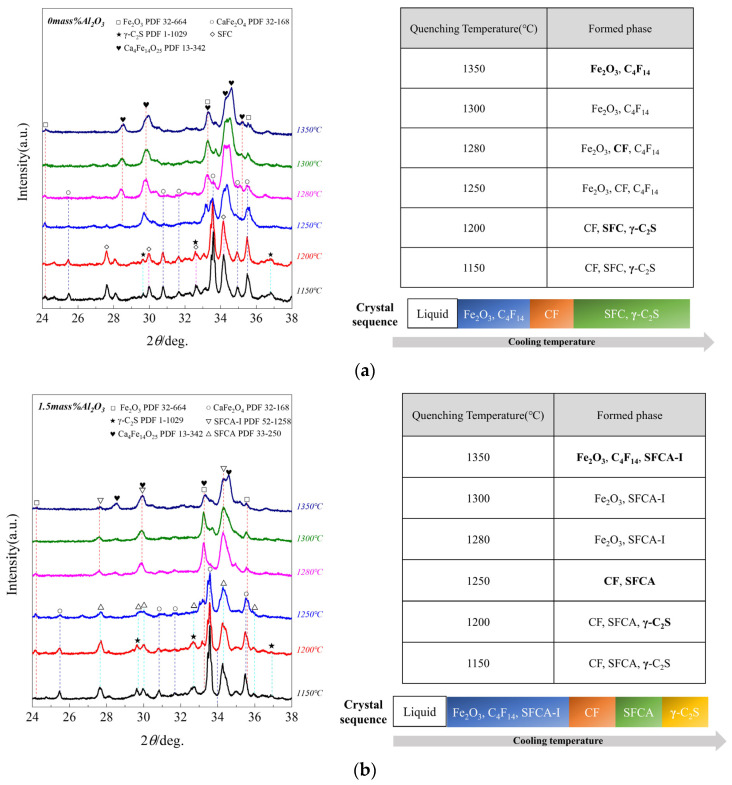

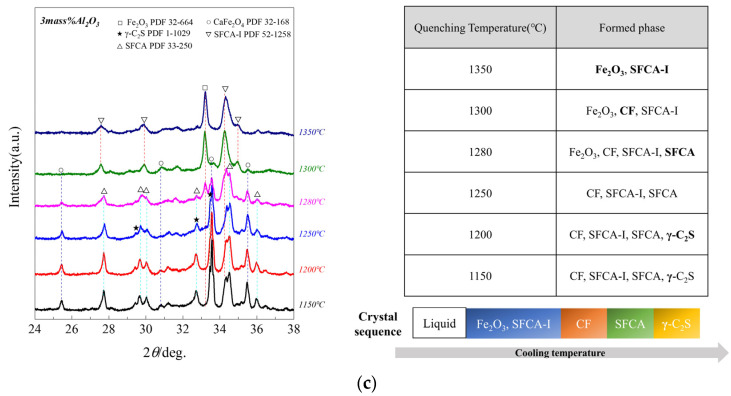
XRD patterns of water-cooled (65 °C/s) samples at different target temperatures (**a**) 0mass% Al_2_O_3_; (**b**) 1.5mass% Al_2_O_3_; (**c**) 3mass% Al_2_O_3_. (The bold contents were the first formed phases in the corresponding temperature).

Due to the strong crystallization ability of calcium ferrite, the crystallization order in the liquid phase cooling process has yet to be understood. For obtaining the sequence of various phases crystallized in the Fe_2_O_3_-CaO-SiO_2_-Al_2_O_3_ system, samples of No.1, No.6, and No.9 (0, 1.5, and 3.0 Al_2_O_3_ mass pct) were selected to further research as cooled to the different target temperature at a cooling rate of 0.02 °C/s, and followed by water cooling. Figure 5 depicts the XRD patterns of the collected samples. It shows that adding Al_2_O_3_ inhibited the formation of C_4_F_14_ and SFC, while it promoted the formation of Fe_2_O_3_, CF, and γ-C_2_S simultaneous to the transformation of SFCA-I into SFCA. The detailed result is as follows:

(1) When Al_2_O_3_ was not added, the crystalline phase of quenched samples was Fe_2_O_3_ and C_4_F_14_ at 1350 °C, while Fe_2_O_3_ and C_4_F_14_ increased at 1300 °C, simultaneously CF appeared; Fe_2_O_3_ and C_4_F_14_ increased at 1280 °C, while Fe_2_O_3_ decreased. CF increased at 1250 °C, simultaneously SFC and γ-C_2_S appeared while Fe_2_O_3_ disappeared; CF increased at 1200 °C, and CF, SFC, and γ-C_2_S increased at 1150 °C. So it can be considered the crystallization sequence was (Fe_2_O_3_, C_4_F_14_) → CF → (SFC, γ-C_2_S).
(2) When Al_2_O_3_ reached 1.5 mass pct, the crystalline phase of quenched samples was Fe_2_O_3_ and C_4_F_14_ at 1350 °C; SFCA-I appeared, simultaneously Fe_2_O_3_ increased at 1300 °C, but C_4_F_14_ disappeared; Fe_2_O_3_ and SFCA-I increased at 1280 °C; SFCA and CF appeared at 1250 °C, simultaneously Fe_2_O_3_ and SFCA-I decreased. At this time, SFCA was formed by the preferentially precipitated SFCA-I and Al^3+^ and Si^4+^ in the melt; CF and SFCA increased at 1200 °C, simultaneously γ-C_2_S appeared; CF, SFCA, and γ-C_2_S increased at 1150 °C. So it can be considered the crystallization sequence was (Fe_2_O_3_, C_4_F_14_) → SFCA-I → CF → SFCA → γ-C_2_S.
(3) When Al_2_O_3_ reached 3.0 mass pct, the crystalline phase of quenched samples was Fe_2_O_3_ and SFCA-I at 1350 °C; SFCA-I increased while Fe_2_O_3_ decreased, simultaneously CF appeared at 1300 °C; Fe_2_O_3_ and SFCA-I decreased at 1280 °C while CF increased, simultaneously SFCA appeared; CF and SFCA increased at 1250 °C while SFCA-I decreased, simultaneously Fe_2_O_3_ disappeared; SFCA-I decreased at 1200 °C while CF and SFCA increased, simultaneously γ-C_2_S appeared; SFCA-I decreased at 1150 °C while CF, SFCA, and γ-C_2_S increased. So it can be considered the crystallization sequence was (Fe_2_O_3_, SFCA-I) → CF → SFCA → γ-C_2_S.

Thermodynamically, the Gibbs free energy of formation of C_2_S is lower than that of CF [27], and the reactions are as Equations (1) and (2), respectively. It shows that C_2_S is more stable to form easier than CF. However, since the added SiO_2_ content of 4mass pct is much smaller than Fe_2_O_3_, resulting in the probability of Si^4+^ reacting with Ca^2+^ is relatively small, simultaneously Si^4+^ also participates in the formation of SFC, SFCA-I, and SFCA. Therefore, the crystallization sequence of C_2_S was late. Since the Gibbs free energy of C_4_F_14_, SFC, SFCA-I, and SFCA formations are not existing in the thermodynamic database, unfortunately it cannot be compared with other crystalline phases.

(1) $$2\mathrm{CaO}+{\mathrm{SiO}}_{2}=2\mathrm{CaO}\cdot {\mathrm{SiO}}_{2}\;\Delta_{f}G_{m}^{\theta }=-118899-11.3T\;(J/\mathrm{mol})$$

(2) $$\mathrm{CaO}+{\mathrm{Fe}}_{2}O_{3}=\mathrm{CaO}\cdot {\mathrm{Fe}}_{2}O_{3}\;\Delta_{f}G_{m}^{\theta }=-29700-4.81T\;(J/\mathrm{mol})$$

From the crystallization order of different samples containing Al_2_O_3_, a new generation path of SFCA-I was found in the crystallization process of the Fe_2_O_3_-CaO-SiO_2_-Al_2_O_3_ quaternary system. The C_4_F_14_ reacts with Si^4+^ and Al^3+^ in the melt to form SFCA-I (C_4_F_14_ + Si^4+^ + Al^3+^ → SFCA-I), and SFCA-I reacts with Si^4+^ and Al^3+^ to form SFCA (SFCA-I + Si^4+^ + Al^3+^ → SFCA).

**Figure 6:**
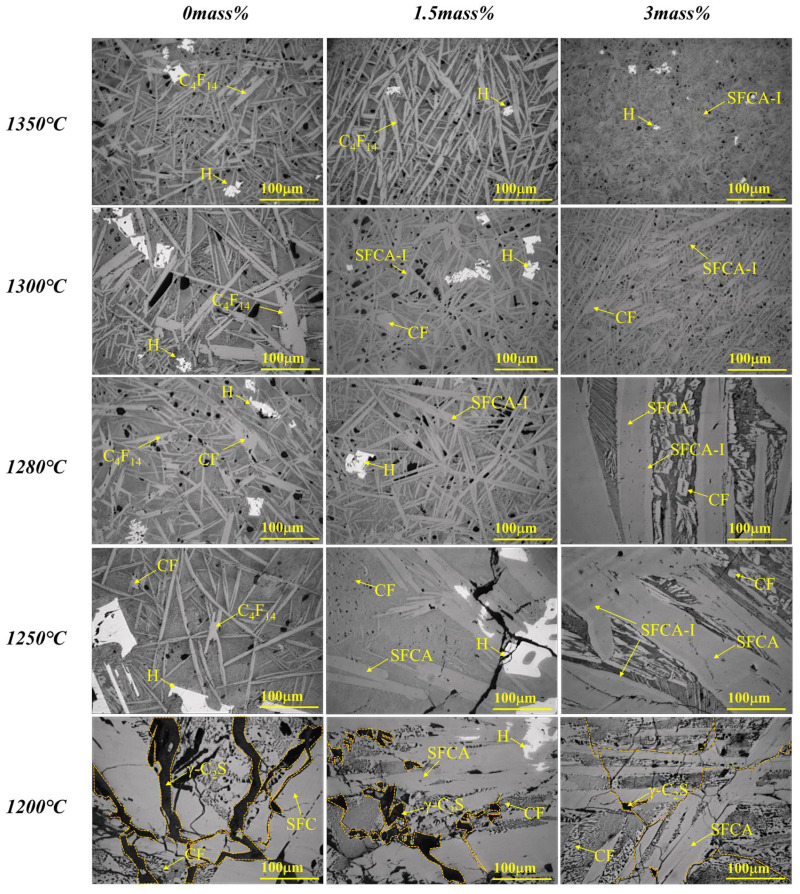
Optical micrographs of cross-sections of water-cooled samples with Al_2_O_3_ addition at different temperatures. CF: CaFe_2_O_4_; C_4_F_14_(Ca_4_Fe_14_O_25_); SFC: Ca_2.73_${\mathrm{Fe}}_{0.04}^{2+}{\mathrm{Fe}}_{10.56}^{3+}$Si_0.66_O_28_; SFCA-I: Ca_3.18_Fe_15.48_Al_1.34_O_36_; SFCA: Ca_5_Si_2_(Fe Al)_18_O_36_; H: Fe_2_O_3_; γ-C_2_S: γ-Ca_2_SiO_4_.

Figure 6 shows the corresponding cross-sectional optical micrograph, where the experimental results are consistent with the XRD results. Seven phases of Fe_2_O_3_, C_4_F_14_, CF, SFC, γ-C_2_S, SFCA-I, and SFCA were co-precipitated in the melt. When the quenched temperature was lowered, equivalent to prolonging the crystallization time, the Fe_2_O_3_ grew up in a lump, and calcium ferrite (CF, C_4_F_14_, SFC) developed from strip to short column. Simultaneously needle-shaped SFCA-I transformed into column-shaped SFCA, and γ-C_2_S developed from block to strip. γ-C_2_S was generated at 1200 °C while degraded when lowered to 1150 °C.

**Figure 7:**
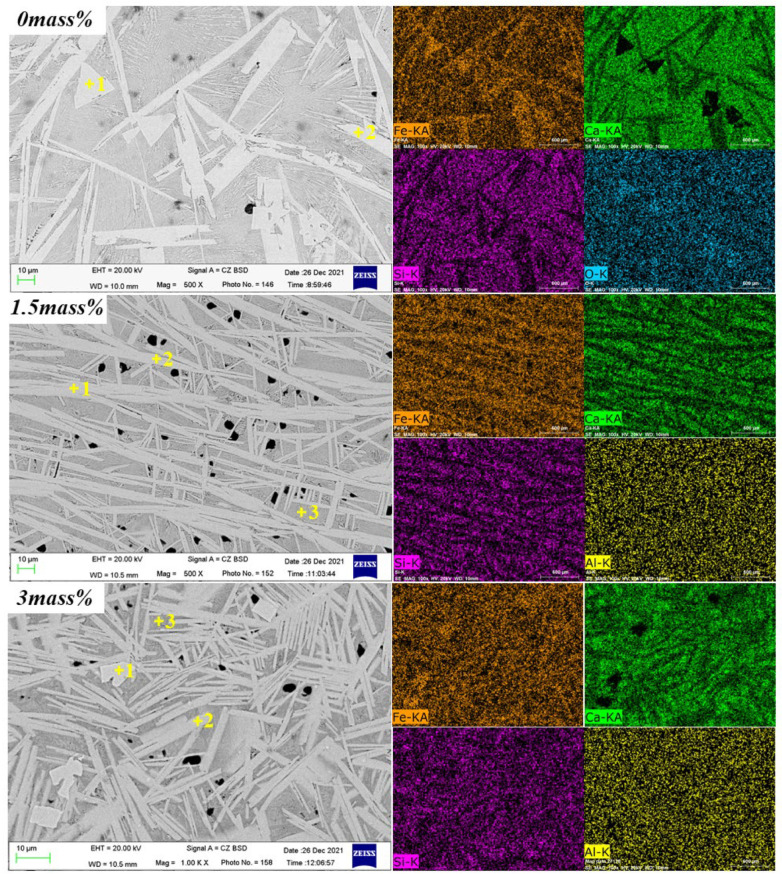
SEM-EDS mapping of the samples, morphology, and distribution of main elements.

Figure 7 and Table 3 show the SEM photos and EDS results of the samples, confirming the XRD results. With the increase of the mass percentage of Al_2_O_3_ in the melt, the crystalline phase would change, and Al_2_O_3_ promoted the transition from SFCA-I to SFCA while inhibiting the formation of C_4_F_14_.

### 3.3. Effect of Cooling Rate on Crystallization

**Figure 8:**
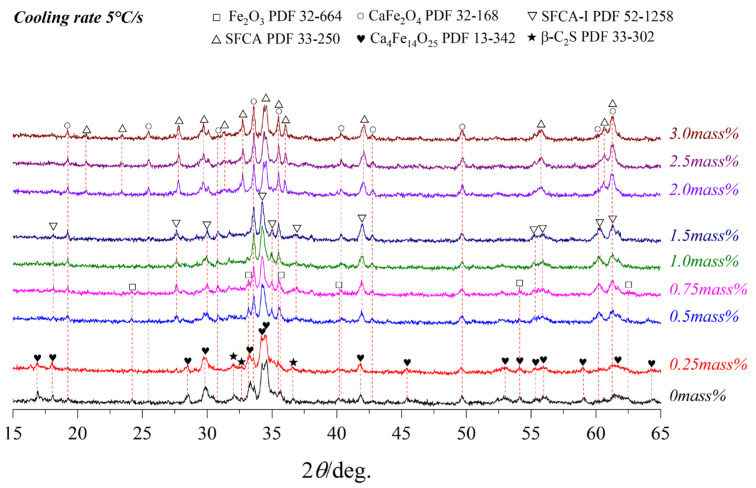
XRD patterns of samples with different Al_2_O_3_ content cooled at 5 °C/s.

**Figure 9:**
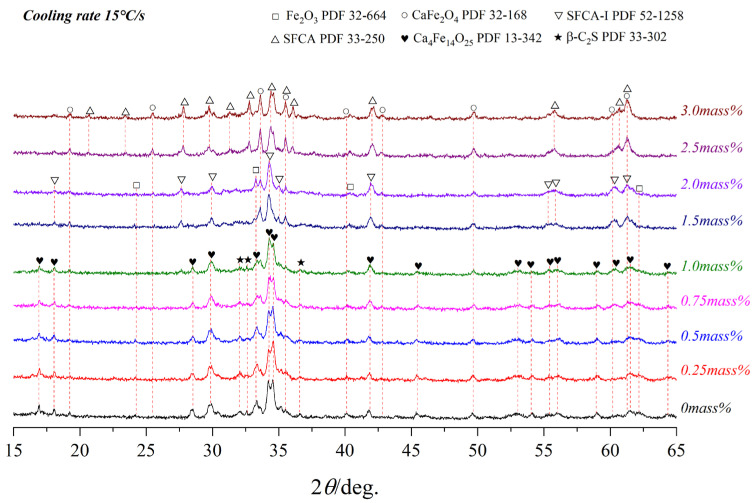
XRD patterns of samples with different Al_2_O_3_ content cooled at 15 °C/s.

**Figure 10:**
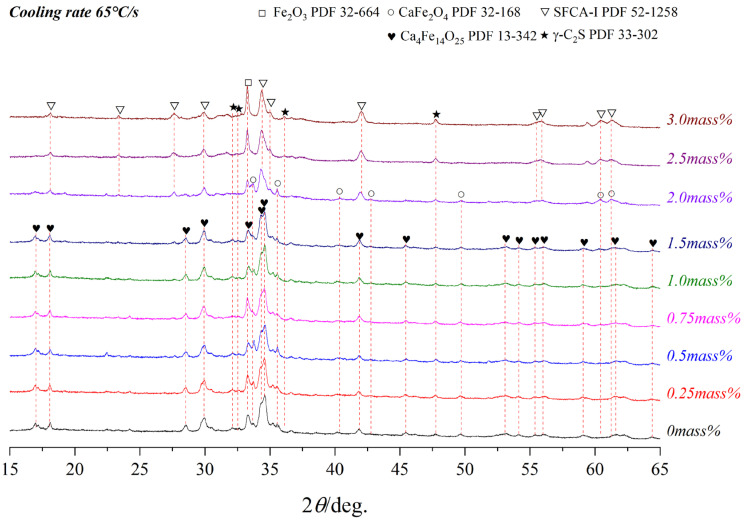
XRD patterns of samples with different Al_2_O_3_ content cooled at 65 °C/s.

**Figure 11:**
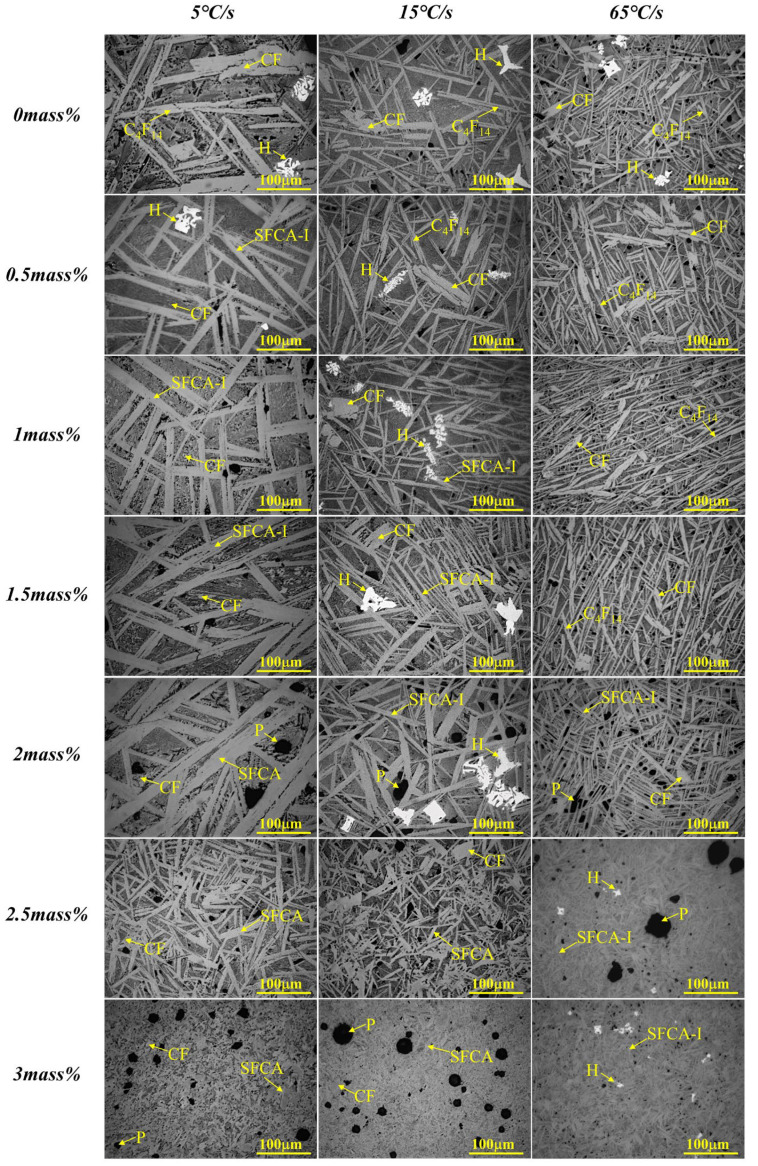
Optical micrographs of cross-sections of crystalline samples with Al_2_O_3_ addition at various cooling rates. C_4_F_14_: (Ca_4_Fe_14_O_25_); CF: CaFe_2_O_4_; SFCA-I: Ca_3.18_Fe_15.48_Al_1.34_O_36_; SFCA: Ca_5_Si_2_(Fe Al)_18_O_36_; H: Fe_2_O_3_; P: Pore.

Figure 8, Figure 9 and Figure 10 present the variations of XRD profiles of crystallized samples with different Al_2_O_3_ content under the cooling rates of 5 °C/s, 15 °C/s, and 65 °C/s, respectively. The corresponding optical micrographs of crystallized phases are presented in Figure 11. Table 4 shows six phases as CF, C_4_F_14_, SFCA-I, SFCA, Fe_2_O_3_, and β-C_2_S.

It can be seen that CF, C_4_F_14_, and Fe_2_O_3_ were generated without Al_2_O_3_. With the increase of the Al_2_O_3_ content, the crystallographic phase transformed significantly as follows. C_4_F_14_ had gradually decreased as C_4_F_14_ reacted with Al^3+^ and Si^4+^ to form columnar SFCA-I. With further increasing the content of Al_2_O_3_, SFCA-I transformed to SFCA. Compared with SFCA, SFCA-I had a higher ratio of Fe_2_O_3_ to CaO in chemical composition. The crystallization of C_4_F_14_ and Fe_2_O_3_ was promoted during the transformation. Moreover, with the increase of Al_2_O_3_ content, the complex calcium ferrite first increased and then decreased. The two-dimensional crystal morphology of the minerals shows that CF was skeletal or corroded. Meanwhile, C_4_F_14_, Fe_2_O_3_, SFCA-I, and SFCA were existed in the morphology of strip, irregular block, column and needle, and short column, respectively.

It can be found that as increasing the cooling rate it shortens the crystal growth time, so the crystalline of some minerals would be inhibited, while the crystalline phase and morphology were also changed significantly. On the one hand, the crystal size would be narrowed. On the other hand, the formation of complex calcium ferrite and the conversion of SFCA-I to SFCA would be promoted, while the formation of SFC and γ-C_2_S would be inhibited. It also promoted the formation of C_4_F_14_, Fe_2_O_3_, and the amorphous phase that filled around the complex calcium ferrite in an imperfect crystallization state. But when the cooling rate reaches 65 °C/s, it was found that C_4_F_14_ was easier to form than Fe_2_O_3_, so C_4_F_14_ should be crystallized with Fe_2_O_3_ first in the crystallization sequence. Therefore, the crystallization order of samples in the Fe_2_O_3_-CaO-SiO_2_-Al_2_O_3_ melt containing Al_2_O_3_ should be C_4_F_14_ → Fe_2_O_3_ → SFCA-I → CF → SFCA → γ-C_2_S.

**Figure 12:**
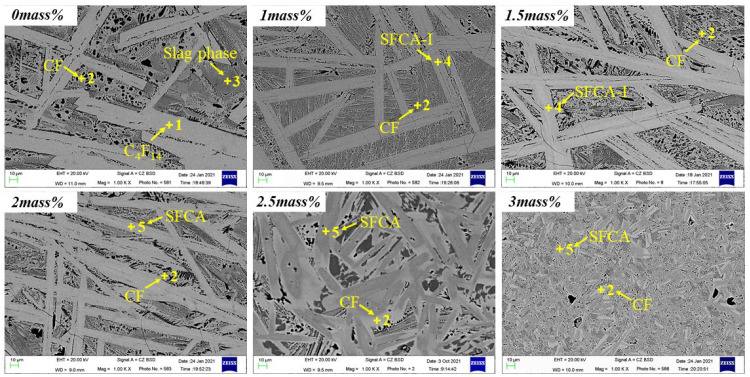
SEM image of the cross-section of the sample after cooling at 5 °C/s.

To further confirm the phase composition, the SEM-EDS analysis of the sample with a cooling rate of 5 °C/s is shown in Figure 12 and Table 5. When the Al_2_O_3_ is not added, only C_4_F_14_ and CF phases were formed. With Al_2_O_3_ content increasing, C_4_F_14_, CF, SFCA-I, and SFCA appeared, which confirmed the experimental results in Figure 8.

**Figure 13:**
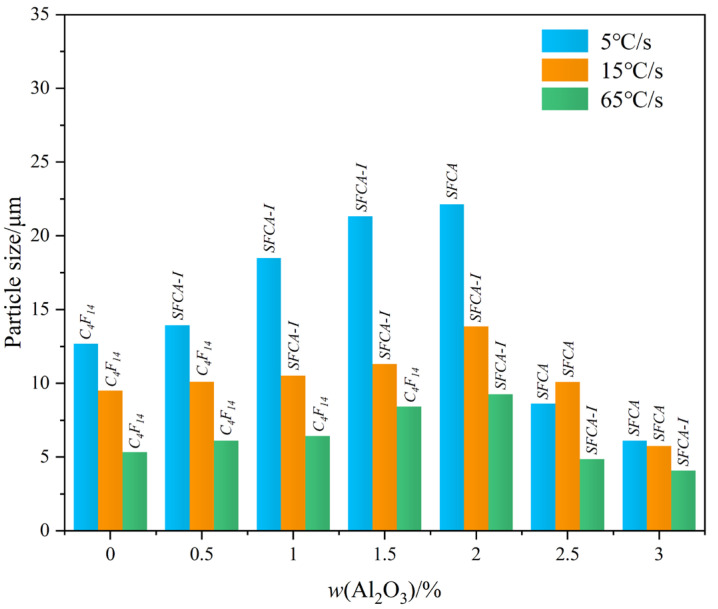
Crystal size of calcium ferrite (C_4_F_14_, SFCA-I, SFCA) at different cooling rates.

To investigate the influence of cooling rate on the morphology of calcium ferrite (C_4_F_14_, SFCA-I, and SFCA) in the Fe_2_O_3_-CaO-SiO_2_-Al_2_O_3_ melt under different Al_2_O_3_ content, the grain size of calcium ferrite in each sample in Figure 11 was measured using the Nano Measurer 1.2 software [29]. Thirty positions in each sample were selected, measured, and an averaged value was calculated. Figure 13 illustrates that with the increased cooling rate, the crystal size of calcium ferrite decreased significantly. Furthermore, when the Al_2_O_3_ content increased, the crystal size of calcium ferrite increased and subsequently decreased, which demonstrates that adding a small amount of Al_2_O_3_ promoted the formation of complex calcium ferrite. At different cooling speeds (5 °C/s, 15 °C/s, and 65 °C/s), the grain size achieved the maximum value (corresponding to 22.15 μm, 13.85 μm, and 9.25 μm, respectively) when the Al_2_O_3_ reached 2.0 mass pct. After Al_2_O_3_ reached 2.5 mass pct, it would increase a viscosity of the melt, which could be a primary reason for the decrease in the crystal size of calcium ferrite.

### 3.4. Discussion on Crystallization Mechanism

To further explain the effect of Al_2_O_3_ on the crystallization of calcium ferrite, FactSage 8.2 software was used to perform thermodynamic equilibrium calculations on the Fe_2_O_3_-CaO-SiO_2_-Al_2_O_3_ system, even though C_4_F_14_, SFCA-I, and SFCA are lacking in the thermodynamic database. Future metallurgical workers are required to improve it.

**Figure 14:**
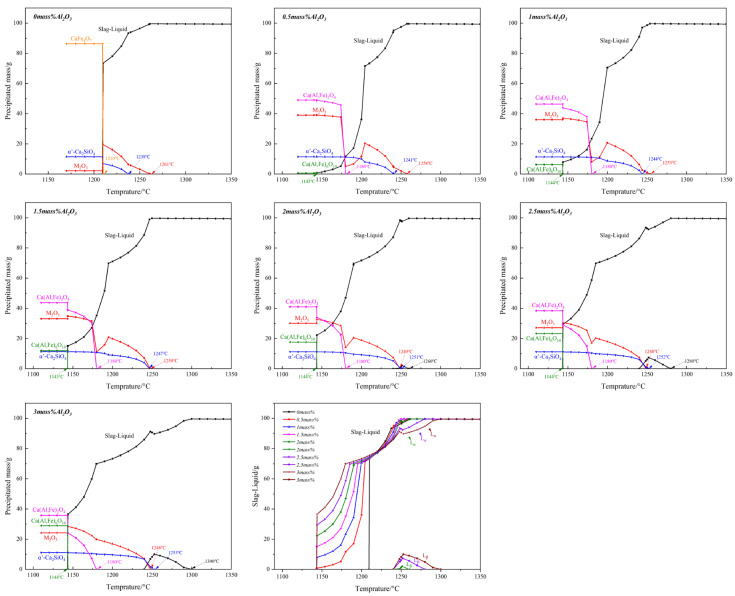
Theoretical crystal phase composition of Fe_2_O_3_-CaO-SiO_2_-Al_2_O_3_ slag during cooling.

Figure 14 and Table 6 show the primary crystallization temperature and crystallization amount of the thermodynamic equilibrium phase with different Al_2_O_3_ content. The result shows that without adding Al_2_O_3_, the phases are M_2_O_3_ (≥99.50 mass pct Fe_2_O_3_ and ≤0.50 mass pct Al_2_O_3_), α’-Ca_2_SiO_4_(α’-C_2_S), and CaFe_4_O_7_(CF_2_). With the Al_2_O_3_ content increasing, CF_2_ disappeared while CF appeared. When Al_2_O_3_ content reached 2.0 mass pct, the primary crystallization phase transformed from M_2_O_3_ to α’-C_2_S, and the transition temperature was 1250 °C. When Al_2_O_3_ content reached 3.0 mass pct, CF and Ca(Al, Fe)_6_O_10_ appeared.

The corresponding crystallization amounts of M_2_O_3_, α’-C_2_S, and CF decreased from 39.01 to 24.19 mass pct, 11.41 to 11.12 mass pct, and 49.00 to 35.76 mass pct, respectively. Ca (Al, Fe)_6_O_10_ increased from 0.579 to 28.924 mass pct.

As shown in Figure 11, many spherical holes appeared in samples when the Al_2_O_3_ content reached 2.5 and 3.0 mass pct, which increased with the increase of the Al_2_O_3_ content and the cooling rate. Simultaneously, the crystalline size of minerals decreased. It can be considered that the increase of the melt viscosity resulted in a slow crystalline rate due to the hard mass transferring.

**Figure 15:**
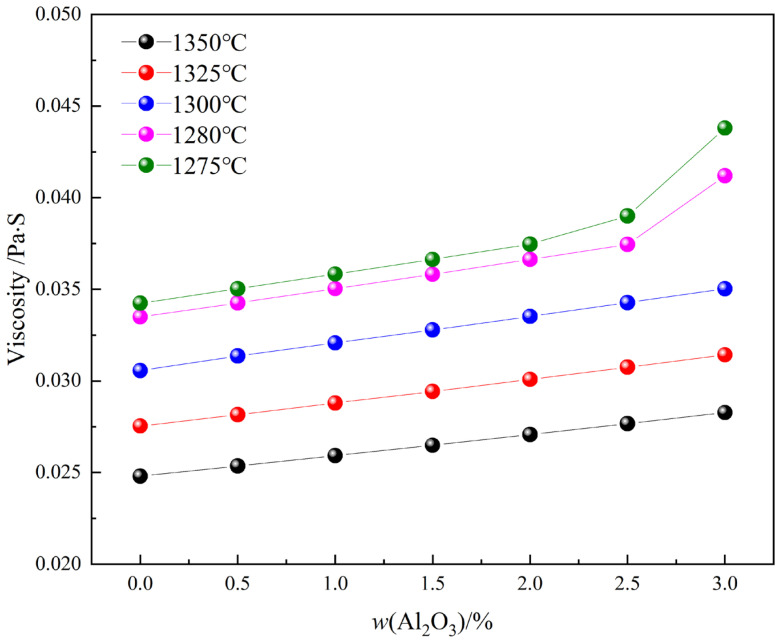
Viscosity diagrams of samples with Al_2_O_3_ addition at different temperatures.

Figure 15 shows the viscosity diagrams of Fe_2_O_3_-CaO-SiO_2_-Al_2_O_3_ melts with Al_2_O_3_ content at different temperatures, which is calculated using thermodynamical software, simultaneously combined by the Einstein-Roscoe formula (Equation (3)) [30], where the mass fraction of the solid phase was obtained as shown in Figure 14. The result shows that at all temperatures, the viscosity value increased with the increase of Al_2_O_3_ content. The viscosity value increased obviously. The viscosity increase would hinder the crystallization and mass transfer of complex calcium ferrite in the melt, resulting in poor crystalline morphology. Specially, after Al_2_O_3_ reached 2.5 mass pct, the viscosity of the melt increased sharply, which could be the main reason for the decrease in the crystal size of calcium ferrite.

(3) $$\eta =\;\eta^{0}\;{\left(1-c\right)}^{-2.5}$$

*η*—solid-liquid mixing viscosity;
*η*^0^—viscosity of pure liquid phase;
c—a mass fraction of solid phase.

**Figure 16:**
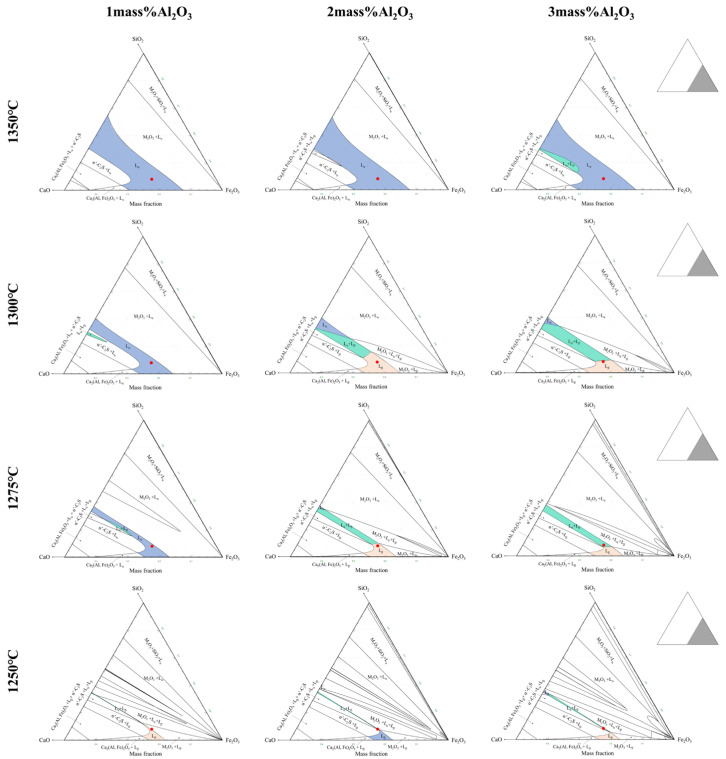
Isothermal cross-section of Fe_2_O_3_-CaO-SiO_2_-Al_2_O_3_ system at different temperatures.

The isothermal cross-sections of Fe_2_O_3_-CaO-SiO_2_-Al_2_O_3_ systems with varying Al_2_O_3_ content at different temperatures are shown in Figure 16. With the Al_2_O_3_ content increasing, the liquid phase is divided into three regions, named L_α_, L_α_ + L_β,_ and L_β_, where the content of Al_2_O_3_ increased from 1.0 to 3.0 mass pct. When the red component point is at 1250 °C, the primary crystal region is transformed from L_α_ + M_2_O_3_ to L_α_ + L_β_ + α’-C_2_S. There may be two reasons for the deterioration of crystallization. On the one hand, the viscosity of L_α_ + L_β_ + α’-C_2_S is higher than that of L_α_+M_2_O_3_. In addition, the crystallization of α’-C_2_S leads to the reducing of initial Ca^2+^ and Si^4+^ in the melt, which is not conducive to the crystallization of complex calcium ferrite. It can be seen from Figure 16 that when Al_2_O_3_ is 2.0 mass pct, not only a certain amount of liquid phase is retained, but also Ca^2+^ and Si^4+^ are not reduced too much. This also explains that when Al_2_O_3_ was 2.0 mass pct, the crystal size of calcium ferrite was the largest, as shown in Figure 13.

## 4. Conclusions

In this work the influences of Al_2_O_3_ content, cooling rate and the crystallization sequence of the Fe_2_O_3_-CaO-SiO_2_-Al_2_O_3_ system during the cooling process were investigated. On this basis, the influence mechanism of Al_2_O_3_ content and cooling rate on the crystallization of complex calcium ferrite (C_4_F_14_, SFCA-I, SFCA) was also proposed. The main conclusions are as follows:

(1) Al_2_O_3_ has an important effect on the composition of the crystal phase of the Fe_2_O_3_-CaO-SiO_2_-Al_2_O_3_ system. Adding alumina promoted the crystallization of Fe_2_O_3_, γ-C_2_S, SFCA-I, and SFCA, while it inhibited the crystallization of C_4_F_14_ and SFC. However, the content of CF first decreased and then increased. This is mainly because of the formation of complex calcium ferrite and the transformation of SFCA-I to SFCA.
(2) The crystallization sequence in Fe_2_O_3_-CaO-SiO_2_-Al_2_O_3_ melt under different Al_2_O_3_ content was investigated, where the corresponding crystalline order is (Fe_2_O_3_, C_4_F_14_) → CF → (SFC, γ-C_2_S), (Fe_2_O_3_, C_4_F_14_) → SFCA-I → CF → SFCA → γ-C_2_S, and (Fe_2_O_3_, SFCA-I) → CF → SFCA → γ-C_2_S under the Al_2_O_3_ content of 0 mass pct, 1.5 mass pct, and 3.0 mass pct respectively. It can be concluded that the C_4_F_14_ reacts with Si^4+^ and Al^3+^ in the melt to form SFCA-I (C_4_F_14_ + Si^4+^ + Al^3+^ → SFCA-I), and then SFCA-I reacts with Si^4+^ and Al^3+^ to form SFCA (SFCA-I + Si^4+^ + Al^3+^ → SFCA).
(3) As the cooling rate increase, C_4_F_14_, SFCA-I, Fe_2_O_3_, β-C_2_S, and the amorphous phases are increased while CF and SFCA are reduced, and the crystal transformation from β-C_2_S to γ-C_2_S can be effectively inhibited. However, when the cooling rate was increased from 15 °C/s to 65 °C/s, C_4_F_14_ was found to crystallize before Fe_2_O_3_.
(4) The crystal size of complex calcium ferrite first increases and then decreases by an increase of Al_2_O_3_ content, and the order of crystal morphology evolved is from the strip, columnar to needle-shaped. When Al_2_O_3_ content reached or exceeded 2.5 mass pct, the viscosity of Fe_2_O_3_-CaO-SiO_2_-Al_2_O_3_ melt increased sharply, resulting in the decrease in the crystalline size of calcium ferrite.

## Figures and Tables

**Table 1:** Chemical Compositions of samples (mass pct).

Samples	Fe_2_O_3_	CaO	SiO_2_	Al_2_O_3_	LT (°C)
No. 1	75.64	20.36	4	0	1261
No. 2	75.45	20.31	3.99	0.25	1259
No. 3	75.26	20.26	3.98	0.50	1257
No.4	75.07	20.21	3.97	0.75	1256
No.5	74.88	20.16	3.96	1.00	1254
No.6	74.51	20.05	3.94	1.50	1250
No.7	74.13	19.95	3.92	2.00	1256
No.8	73.75	19.85	3.90	2.50	1276
No.9	73.37	19.75	3.88	3.00	1293

**Table 2:** EDS results of the crystallized phases in the samples under 0.02 °C/s.

Sample No.	Marked Points	Crystal Phase	Elements (at Pct)				
			Fe	Ca	Si	Al	O
0mass%	P_1_	C_4_F_14_	46.20	13.54	1.79	0.74	37.72
	P_2_	CF	39.38	21.93	1.38	0.94	36.37
	P_3_	γ-C_2_S	0.61	40.07	20.33	0.24	38.75
	P_4_	Fe_2_O_3_	62.63	0.06	1.45	0.95	34.91
1.0mass%	P_2_	CF	41.42	23.82	0.00	0.10	34.67
	P_3_	γ-C_2_S	0.48	41.01	19.91	0.31	38.29
	P_4_	Fe_2_O_3_	67.98	0.14	0.42	0.91	30.54
	P_5_	SFCA-I	47.64	14.79	2.42	2.15	33.01
1.5mass%	P_2_	CF	38.51	22.76	0.31	0.29	38.12
	P_3_	γ-C_2_S	0.86	37.95	17.98	0.26	42.96
	P_4_	Fe_2_O_3_	64.33	0.27	0.00	0.72	34.67
	P_5_	SFCA-I	43.01	14.34	3.83	3.65	35.16
2.0mass%	P_2_	CF	41.02	24.77	1.40	1.32	31.49
	P_3_	γ-C_2_S	0.64	42.53	21.15	0.35	35.33
	P_4_	Fe_2_O_3_	68.94	0.00	0.04	0.92	30.10
	P_5_	SFCA-I	47.06	14.56	3.20	3.20	31.98
2.5mass%	P_2_	CF	39.31	24.09	0.01	0.28	36.31
	P_3_	γ-C_2_S	0.35	39.08	19.78	0.16	40.63
	P_4_	Fe_2_O_3_	62.79	0.28	0.00	0.84	36.09
	P_6_	SFCA	41.35	13.26	3.83	5.00	36.56
3.0mass%	P_2_	CF	41.32	26.74	0.00	0.40	31.53
	P_3_	γ-C_2_S	0.94	45.13	17.11	0.00	36.82
	P_6_	SFCA	42.86	13.37	4.17	7.19	32.41

**Table 3:** EDS results of the crystallized phases in the samples with different mass pct of Al_2_O_3_.

Sample No.	Marked Points	Phase	Elements				
			Fe	Ca	Si	Al	O
0mass%	P_1_	Fe_2_O_3_	68.63	0.49	1.36	0	28.94
	P_2_	C_4_F_14_	51.13	14.23	1.16	0	33.48
	P_3_	Slag phase	35.81	22.88	5.90	0	35.41
1.5mass%	P_1_	C_4_F_14_	47.25	12.37	0.00	1.08	39.30
	P_2_	SFCA-I	44.04	12.68	1.08	2.20	39.99
	P_3_	Slag phase	44.52	15.88	2.71	2.55	34.34
3.0mass%	P_1_	Fe_2_O_3_	54.96	0.62	0.00	1.10	43.32
	P_2_	SFCA-I	39.52	11.80	2.31	4.84	41.53
	P_3_	Slag phase	36.85	15.91	3.18	4.12	39.95

**Table 4:** The crystalline phase of the corresponding samples in Figure 8, Figure 9 and Figure 10.

Cooling Rate (°C/s)	Al_2_O_3_ (Mass Pct)						
	0	0.5	1	1.5	2	2.5	3
5	CF	CF	CF	CF	CF	CF	CF
	C_4_F_14_	SFCA-I	SFCA-I	SFCA-I	SFCA	SFCA	SFCA
	Fe_2_O_3_	Fe_2_O_3_					
	β-C_2_S						
15	CF	CF	CF	CF	CF	CF	CF
	C_4_F_14_	SFCA-I	SFCA-I	SFCA-I	SFCA-I	SFCA-I	SFCA-I
	Fe_2_O_3_	Fe_2_O_3_	Fe_2_O_3_	Fe_2_O_3_	Fe_2_O_3_	Fe_2_O_3_	Fe_2_O_3_
	β-C_2_S	β-C_2_S	β-C_2_S				
65	CF	CF	CF	CF	CF		
	C_4_F_14_	C_4_F_14_	C_4_F_14_	C_4_F_14_	SFCA-I	SFCA-I	SFCA-I
	Fe_2_O_3_			Fe_2_O_3_	Fe_2_O_3_	Fe_2_O_3_	Fe_2_O_3_
	β-C_2_S	β-C_2_S	β-C_2_S	β-C_2_S	β-C_2_S	β-C_2_S	β-C_2_S

**Table 5:** The EDS results of the corresponding marked points in Figure 12.

Al_2_O_3_ (Mass Pct)	Marked Points	Phase	Elements (at Pct)				
			Fe	Ca	Si	Al	O
0	P_1_	C_4_F_14_	52.06	12.37	0.79	0	34.78
	P_2_	CF	40.74	19.59	3.96	0	35.72
	P_3_	Slag phase	37.10	20.93	6.93	0	35.04
1.0	P_2_	CF	40.49	21.60	4.95	1.77	31.20
	P_4_	SFCA-I	48.28	14.24	2.09	0.92	34.48
1.5	P_2_	CF	42.16	19.75	4.89	2.06	31.14
	P_4_	SFCA-I	49.19	14.77	2.94	2.77	30.33
2.0	P_2_	CF	43.43	26.11	0	0.69	29.77
	P_5_	SFCA	47.35	14.22	3.40	4.42	30.62
2.5	P_2_	CF	39.39	21.58	4.51	3.94	30.58
	P_5_	SFCA	43.77	12.73	4.28	6.27	32.95
3.0	P_2_	CF	39.39	21.58	4.51	3.94	30.58
	P_5_	SFCA	40.10	13.17	4.26	6.25	36.22

**Table 6:** Changes of primary crystallization phase and temperature after adding Al_2_O_3_.

Al_2_O_3_ Content (Mass Pct)	Primary Phase	Primary Crystallization Temperature (°C)
0	M_2_O_3_	1261
0.5	M_2_O_3_	1258
1.0	M_2_O_3_	1253
1.5	M_2_O_3_	1250
2.0	α’-C_2_S	1251
2.5	α’-C_2_S	1252
3.0	α’-C_2_S	1253

## Data Availability

Data are contained within the article.
